# Eplet mismatch analysis and allograft outcome across racially diverse groups in a pediatric transplant cohort: a single-center analysis

**DOI:** 10.1007/s00467-019-04344-1

**Published:** 2019-10-10

**Authors:** Mary Carmelle Philogene, Anita Amin, Sheng Zhou, Olga Charnaya, Renato Vega, Niraj Desai, Alicia M. Neu, Cozumel S. Pruette

**Affiliations:** 1 Department of Medicine, Johns Hopkins School of Medicine, 2041 E. Monument Street, Baltimore, MD 21205 USA; 2 Immunogenetics Laboratory, 2041 E. Monument Street, Baltimore, MD 21205 USA; 3 Department of Neurology, University of North Carolina School of Medicine, 115 Mason Farm Road, Chapel Hill, NC 27599 USA; 4 Department of Surgery, Johns Hopkins University School of Medicine, 720 Rutland Ave Turner 34, Baltimore, MD 21205 USA; 5 Department of Epidemiology, Johns Hopkins School of Public Health, Baltimore, MD USA; 6 Department of Pediatric Nephrology, Rubenstein Child Health Building, Johns Hopkins University School of Medicine, 200 N. Wolfe Street, Baltimore, MD 21287 USA

**Keywords:** Eplet, Donor, Recipient

## Abstract

HLA eplet mismatch load has been suggested as an improvement to HLA antigen mismatch determination for organ selection. Given that eplet mismatches are determined based on amino acid sequence difference among HLA alleles, and that the frequency of HLA alleles varies between racial groups, we investigated the correlation between eplet mismatch load and allograft outcomes in 110 pediatric kidney transplant recipients who received their first organ from a donor of the same race (SRT) versus a donor of a different race (DRT). Adjusted modified Poisson regression was used to assess the interaction between eplet mismatch load and race mismatch and its effect on outcome. Caucasians and living donor recipients had lower eplet mismatched loads against their donors compared with non-Caucasian and deceased donor recipients. Overall, for the entire population, the risk of de novo HLA-DSA development was significantly increased with higher eplet loads (*p* < 0.001). Compared with the SRT group, the DRT group had higher eplet loads when compared with their donor, for HLA class I but not HLA class II molecules; however, there was no significant difference in the incidence of de novo HLA-DSA between the 2 groups. The risk of rejection increased significantly for DRT compared with SRT, only when class I eplet load was ≥ 70 (*p* = 0.04). Together this data show that eplet mismatch load analysis is an effective tool for alloimmune risk assessment. If considered for donor selection, acceptable eplet mismatch loads determined from studies in homogenous populations may restrict transplantation across racially diverse donor and patient groups with no evidence of poor outcome. Therefore, an acceptable eplet mismatch load threshold must consider the heterogeneity of the transplant population.

**Electronic supplementary material:**

The online version of this article (10.1007/s00467-019-04344-1) contains supplementary material, which is available to authorized users.

## Introduction

Kidney transplantation is considered the treatment of choice for children with end-stage renal disease, as this minimizes the impact of the uremic milieu on neurocognitive development and growth [1–4]. Both “Share 35” and the new kidney allocation system (KAS) have focused on quicker access to deceased kidney donors for pediatric transplant candidates in the USA [5, 6]. These allocation changes have also aimed at allowing pediatric patients to receive the highest quality donors; those less than 35 years old and with a kidney donor profile index of 35% or less, respectively [7]. Shorter time on dialysis and decreased waiting time for a transplant are often considered a priority over HLA matching [4, 6, 8]. However, studies in pediatric kidney transplantation show that poor HLA matching is associated with shorter time to allograft loss, increased use of immunosuppression due to repeated rejection episodes, and difficulty obtaining a second transplant due to development of HLA antibodies [9–11]. These observations have sparked a renewed interest in strategies for better HLA matching, particularly for this age group [12].

**Fig. 1:**
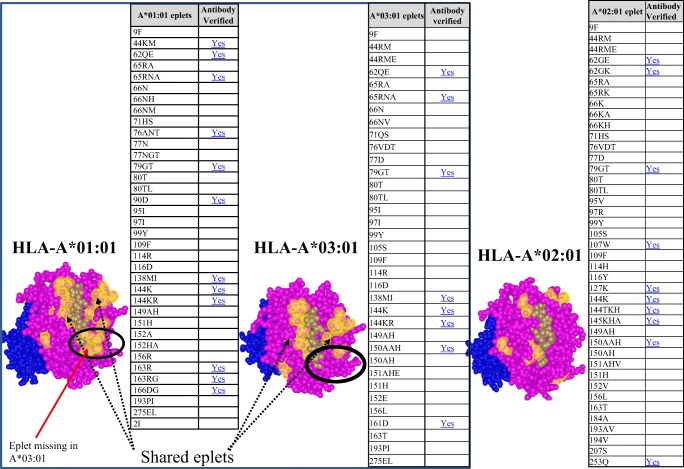
Comparison of crystal structures between HLA-A*01:01, HLA-A*02:01, and HLA-A*03:01. Theoretical structures were produced in HLA Fusion ™ Version 4.2. HLA-A*01:01 and HLA-A*03:01 have shared eplets. Eplets unique to HLA-A*01:01 but not present in HLA-A*03:01 are circled in black. Antibody verified eplets are listed as “YES” in table. HLA-A*02:01 is more distinct from HLA-A*:01:01 and HLA-A*03:01. Pink = alpha domain; blue = beta 2 microglobulin; brown = bound peptide; yellow = eplets

The conventional method for determining HLA compatibility has been to match HLA antigens, reported at the serologic level, between donor and recipient. However, organ allocation based on HLA antigen matching proved to be a disadvantage for racial minority groups who have different HLA antigen frequencies and rare HLA antigens when compared with the donor population [13]. Eplet-based matching has been suggested as a more precise strategy compared with HLA antigen matching [14, 15]. Eplets are clusters of polymorphic amino acids, discontinuous or linear, located on the surface of HLA molecules (Fig. 1). Eplets have been called “functional epitopes” [16, 17] as they include 2 to 3 amino acids that can be recognized by HLA antibodies, among the 15 to 22 amino acids that make up an HLA epitope. The HLAMatchmaker software [18] has been used to determine the number of eplets that are different between a donor and recipient’s HLA phenotypes (eplet mismatch load). This matching program includes eplets that are known to elicit antibody production (antibody verified eplets; Fig. 1), as well as eplets determined theoretically using modeling with crystalized HLA molecules (non-antibody verified eplets; Fig. 1). The combined number of antibody verified and non-verified eplets make up the total eplet load, and the higher the eplet-mismatched load, the greater the incompatibility between donor and recipient. Furthermore, because eplets are shared among several HLA antigens (Fig. 1), eplet-based matching could identify acceptable matches among antigens that may appear to be different serologically. A few studies have evaluated this approach for donor selection in pediatric kidney transplantation, and reported improved outcomes [19, 20].

Given that eplet mismatches are determined based on HLA alleles and that the frequency of HLA alleles varies between racial groups, we sought to investigate whether higher eplet loads between patients and donors of different race results in worst allograft outcome compared with patients and donors of the same race. We compared eplet loads and outcomes such as development of de novo HLA-DSA, incidence of rejection, and allograft loss in pediatric transplant patients who received their first kidney transplant from a donor of the same race (SRT) versus from a donor of a different race (DRT).

## Methods

### Study population

This is a retrospective cohort study of pediatric (≤ 21 years old) kidney transplant recipients followed at the Johns Hopkins Children Center, who received a first kidney transplant between January 2006 and April 2017. In this group, 92 patients were transplanted at Johns Hopkins Hospital and 18 patients were transplanted at other centers prior to follow-up at Johns Hopkins. Patient and donor demographics and clinical information were retrieved from the electronic patient information records (EMR) under an approved IRB protocol.

Donor selection for pediatric candidates listed for transplantation at Johns Hopkins includes an evaluation of the level of HLA mismatch between donor and recipient with the goal of limiting the incidence of antibody development. Transplant candidates are discussed during quarterly meetings between clinicians and the histocompatibility laboratory. Thresholds for acceptable HLA mismatches with a deceased or living donor are established for candidates based on each patient’s clinical characteristics, including the urgency for transplantation, sensitization against HLA antibody (calculated PRA), and the frequency of the patient’s HLA antigens compared with the donor pool. Candidates with several potential living donors who are medically suitable for donation may be paired with the best HLA-matched donor.

### Immunosuppression protocol

All patients transplanted at Johns Hopkins received induction therapy. Induction treatment typically consisted of Thymoglobulin (1.5 mg/kg/day for 5 days; *n* = 79); in a few cases, Daclizumab (1 mg/kg/day for 5 days; *n* = 7), Basiliximab (20 mg at time of transplant and 20 mg on post-operative day 4; *n* = 6) all with methylprednisolone 10 mg/kg delivered at the time of transplant (day 0), followed by 8 mg/kg on day 1, 6 mg/kg on day 2, 4 mg/kg on day 3, 2 mg/kg on day 4, and 1 mg/kg on day 5. Three patients transplanted at other institutions were given Alemtuzumab (standard dose 30 mg at time of transplant) as induction. Maintenance treatment consisted of either steroid free or steroid minimization (0.2 mg/kg/day or qod), mycophenolate mofetil (initially 1200 mg/m^2^/day, with decrease to 600 mg/m^2^/day when tacrolimus therapeutic), and tacrolimus (goal serum level of 7–10 ng/mL). Indications for steroid minimization versus steroid free included underlying immunologic disease and/or presence of HLA antibody.

### Histocompatibility testing

HLA-A, HLA-B, HLA-C, HLA-DR, HLA-DQ, and HLA-DP typing were performed by reverse sequence-specific oligonucleotide probe (SSO) assay (One Lambda LABType®) and HLA antigen equivalents were reported for donors and patients who were transplanted at Johns Hopkins. HLA-typing data from transplants not performed at Johns Hopkins were retrieved from patients’ archived medical records stored in Johns Hopkins EMR or UNOS records. HLA mismatches between recipients and their donor were determined at the HLA-A, HLA-B, HLA-C, HLA-DRB1, HLA-DQB1, and HLA-DPB1 loci and were assigned as 0, 1, or 2 antigen mismatches for each locus. HLA antibody specificities were identified using multianalyte bead-based assays performed on the Luminex® platform (Immucor-Lifecodes, Stamford, CT and One Lambda, Canoga Park, CA). Based on the single antigen beads, levels of antibodies against HLA-A, HLA-B, and HLA-DR were reported as cytotoxic positive for MFI values > 10,000, flow cytometric positive (FCXM+) for MFI values 4000 to < 10,000, or FCXM- Luminex+ (Lum+) for MFI values 2000 to < 4000. Antibodies against HLA-C, HLA-DQ, and HLA-DP were reported for MFI values > 20,000 as CDC+, 16,000 as FCXM+, and 4000 as Lum+. MFI below these values were evaluated based on reactivity patterns on several assays and those that lacked specificity patterns were reported as negative. Cytotoxic crossmatch tests were performed prior to transplantation, with positively selected T and B lymphocyte targets [21]. FCXM tests were performed as needed and as previously described [22] and were acquired on BD FACSCanto II using FACSDIVA software (BD Bioscience, Franklin Lakes, NJ). Removal of interfering substances in patients’ sera, including IgM autoantibodies and IgG immune complexes, was done by hypotonic dialysis [23]. Post-transplantation, HLA antibody measurements were performed in cases where pre-transplant HLA-DSA was present, if serum creatinine rose greater than 20% above the baseline, or if a biopsy indicated dysfunction. A biopsy indicating dysfunction is defined as injury on biopsy with a diagnosis of rejection (cellular mediated, antibody mediated), transplant glomerulopathy, or if a biopsy showed evidence of viral infiltration. Antibodies reported as de novo HLA-DSA were > 1000 MFI and confirmed using multiple assays.

### Eplet mismatch analysis

The HLA Matchmaker software (HLA-ABC version 02, update June 2016 and HLA-DRDQDP (includes DRβ1/3/4/5, DQβ1 and DPβ1), version 2.1, update January 2017) was used to identify recipient-donor mismatches at the eplet level [24, 25]. Evaluation of eplet mismatching was done using the most common allele when an intermediate resolution typing was available for both recipient and donor. For patients and donors with only serologic level typing, the HaploStat application (https://www.haplostats.org) was used to estimate the most likely allele based on the race of the patient and the donor [26, 27]. To further confirm that there was no significant difference between the first and second allele in the string, both were evaluated for eplet load. The total number of eplets for each locus was counted and included antibody verified and non-verified eplets. Data obtained with the HLA Matchmaker software was further verified using One Lambda’s HLA Fusion version 4.2 software which features integration of an HLA Matchmaker module developed in partnership with Dr. Rene Duquesnoy.

### Clinical outcomes

The clinical outcomes were obtained from chart review. Rejections were diagnosed by biopsy performed for creatinine rise, proteinuria, or hematuria and included a review of all clinically indicated allograft biopsies performed during the follow-up time for each patient. Biopsies were graded according to BANF 2009–2013 criteria [28–31]. Graft loss was defined as return to dialysis maintenance or requirement for repeat transplant. Recurrence of disease was defined as any post-transplant recurrent diagnosis of a pre-transplant condition. Viral infection was defined as any diagnosis of EBV or CMV infection reported post-transplant based on clinical findings in the presence of viremia. BK infection was included only if confirmed by findings on biopsy. Medication non-adherence was considered present if concerns about adherence were included in documentation by the members of the health care team.

### Statistical analysis

Analyses were performed in GraphPad Prism (version 6) and Stata/SE 14.1 for Windows (College Station, Texas). Continuous variables were compared using Student’s *t* test or Wilcoxon rank-sum test as appropriate and categorical variables were compared using the *χ*2 test and/or Fisher’s exact test. The relative risks for each outcome based on the number of eplet mismatches at different HLA loci were determined for the entire cohort and for race match status between donor and recipient (SRT vs DRT) using unadjusted and adjusted models. Because there were more than 10% for all tested outcomes, modified Poisson regressions were used to compare interactions [32]. Effect measure modification of eplet mismatch was examined by testing the interactions between eplet mismatch and recipient and donor race match status for each outcome. We adjusted for donor and recipient age (age and age-squared to account for the nonlinearity), gender, donor source, kidney allocation period, recipient diagnosis, and CPRA. A complete case analysis was done, in which observations with missing information on any covariates were excluded from the analysis and *p* values less than 0.05 were considered statistically significant.

## Results

### Characteristics of kidney transplant recipients and donors

Of 155 pediatric kidney transplant patients followed at the Comprehensive Transplant Center at Johns Hopkins between January 2006 and July 2017, 113 patients were first transplant recipients. Three patients for which complete donor HLA typing information was missing were excluded from the study. The characteristics of the remaining 110 first kidney transplant recipients are summarized in Table 1. The mean follow-up time was 5.8 years (0–11 years). The median age at time of transplantation was 13 years (2–21 years old). The transplanted cohort consisted of 60% male and 52% Caucasian recipients. Pre-transplant HLA antibody levels were missing for the patients transplanted at other centers. The majority of patients with available pre-transplant HLA antibody tests (79%) were negative for HLA antibody prior to transplantation and only 5% were transplanted across a Luminex + antibody directed against a donor antigen (HLA-DSA). Overall, there were slightly more living donor (55%) compared with deceased donor (45%) transplants. The number of living-related versus living-unrelated donors was 45(74%) and 16 (26%), respectively. Donors were mostly Caucasian (61%) and male (52%), ages 10 to 49 years old. Despite the reported decrease in kidney donation from living donors after the enactment of Share 35 in 2005 nationally [5], of 98 transplants performed in this cohort, between 2006 and 2014, 56% of the organs were from living donors. The number of deceased donor transplants did not increase significantly during 15 months (January 2015 and April 2017) after the implementation of the new KAS in December 2014 (44% versus 50% for pre and post KAS, respectively; *p* = 0.6).

**Table 1:** Patient and donor characteristics

Pre-transplant patient characteristics	*n* = 110
Male, *n* (%)	67 (60)
Mean age at transplant (range)	13.4 (2–21)
Race, *n* (%)	
Caucasian	57 (52)
African American	38 (34)
Other	15 (14)
Pre-transplant HLA sensitization, *n* (%)	
Pre-transplant CPRA = 0%	87 (79)
Pre-transplant CPRA = 10–50%	4 (3.6)
Pre-transplant CPRA > 50%	1 (0.9)
No information on pre Tx CPRA	18 (16)
Pre-Tx HLA-DSA positive	6 (5)
Primary diagnosis, *n* (%)	
Anoxia/ischemia	8 (7)
ARPKD/ADPKD	2 (2)
CAKUT^1^	36 (33)
Ciliopathy	9 (8)
Cystinosis	1 (0.9)
FSGS	20 (18)
GN	17 (15)
HUS	1 (0.9)
SLE	1 (0.9)
Unclear etiology	11 (10)
Other^2^	4 (4)
Donor characteristics	
Living donor (related and unrelated), *n* (%)	61 (55)
Deceased donor, *n* (%)	49 (45)
Mean donor age (range)	33 (10–49)
Donor race, *n* (%)	
Caucasian	67 (61)
African American	21 (19)
Other	11 (10)
Missing race information	11 (10)
Donor male, *n* (%)	57 (52)
Donor female, *n* (%)	41 (37)
Missing information for donor gender, *n* (%)	12 (11)
No. transplanted per allocation era, *n* (%)	
2006–2014 (Post Share 35)	98 (89)
Deceased donors	43 (44)
Living donors (related and unrelated)	55 (56)
2015–July 2017 (post KAS)	12 (11)
Deceased donors	6 (50)
Living donors (related and unrelated)	6(50)

^1^Congenital anomalies of the kidney and urinary tract

^2^Other causes of end-stage renal disease due to calcineurin inhibitor toxicity, mathylmalonic acidemia, hepatorenal syndrome

### HLA antigen mismatch and eplet mismatch between recipients and their donors

We assessed antigen mismatches by donor source and recipient race based on low-resolution HLA typing. HLA-A, HLA-B, and HLA-DR typing were available for all patients. HLA-C, HLA-DQ, and HLA-DP typing were missing for 5 of 110 (4.5%), 2 of 110 (1.8%), and 28 of 110 (25%) patient/donor pairs. As shown in Table 2, Caucasian recipients had significantly fewer HLA class I mismatches with their donor compared with non-Caucasian patients (*p* = 0.006), but there was no significant difference in HLA-class II antigen mismatches between the racial groups (*p* = 0.126). Patients who received an organ from a deceased donor had significantly more mismatches with their donors at all loci compared with patients who received an organ from a live donor (*p* < 0.001; Table 2).

**Table 2:** Comparison between number of HLA antigen MM by race and donor source

	Class I-MM (of 6 ags^2^)	*p*	Class II-MM (of 6 ags^3^)	*p*	HLA-DR MM (of 2 ags)	*p*	HLA-DQ MM (of 2 ags)	*p*
Recipient race								
Caucasian	3.1	0.006	2.9	0.126	1.0	0.169	0.8	0.199
African American	4.1		3.4		1.2		1.0	
Other race^1^	3.8		3.6		1.3		1.1	
Donor type								
Deceased	4.5	< 0.001	4.2	< 0.001	1.4	< 0.001	1.1	< 0.001
Living	3.0		2.5		0.9		0.7	

^1^Other races include Asian, Hispanic, American Indian, and Mid-Eastern

^2^Includes HLA A,B,C antigens

^3^Includes HLA DR, DQ, DP antigens

Eplet mismatch analysis could be performed for 105 of the 110 patients for HLA class I (A, B, C), 82 of the 110 patients for HLA class II (DRβ1/3/4/5, DQβ1, DPβ1), 110 patients for HLA-DRβ1/3/4/5 only, and 82 patients for HLA-DQβ1 only. Similar to the antigen level mismatches, there were significantly fewer numbers of HLA class I eplet mismatches between Caucasian recipients and their donors compared with all other racial groups (*p* = 0.028; Table 3). There was no significant difference in HLA-DRβ1/3/4/5 (*p* = 0.456) or DQα1/DQβ1 (*p* = 0.397) eplet load based on recipient race. The eplet mismatch load was significantly higher at all loci for recipients of a deceased donor kidney compared with those who were transplanted with a living donor (Table 3). A significant difference was also noted between recipients of a living-related versus living-unrelated donor for class I eplet load (*p* < 0.001), but not for class II eplet load (*p* = 0.052) (Table 3).

**Table 3:** Mean number of eplet MM by race and donor source

	ABC Eplet MM	*p*	DRβ/DQβ/DPβ Eplet MM	*p*	DRβ1 Eplet MM	*p*	DQβ1 Eplet MM	*p*
Recipient race								
Caucasian	28	0.028	24	0.197	14	0.456	8	0.397
African American	37		30		13		10	
Other race^1^	33		27		16		10	
Donor type								
Deceased	40	< 0.001	34	< 0.001	16	0.012	11	0.004
Living	27		21		12		7	
Living related	24	< 0.001	20	0.052	11	0.054	6	0.11
Living unrelated	37		28		16		9	

^1^Other races include Asian, Hispanic, American Indian, and Mid-Eastern

**Fig. 2:**
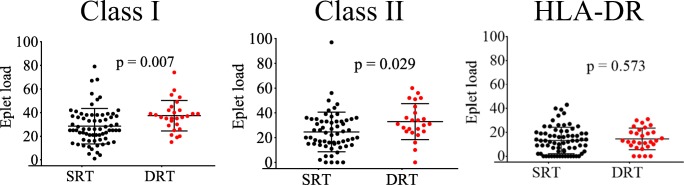
Eplet load difference between SRT and DRT groups. HLA- class I eplet mismatch load (ABC) between donor and recipient in the DRT group (*n* = 29) was higher than that of SRT group (*n* = 70) (mean eplet load = 37 versus 28, respectively; 95% CI 2.4–15.3; *p* = 0.007). HLA-class II eplet load (include HLA-DRβ1/DRβ345/DQβ1/DPβ1) was higher in DRT compared with SRT (33 versus 25, respectively; 95% CI 0.8–15.8; *p* = 0.029). There was no significant difference in the mean eplet load at HLA-DRβ1 for DRT versus SRT (mean eplet loads 13 versus 14, respectively; *p* = 0.573)

The cohort was then grouped into recipients who received a transplant from a donor with same race (SRT; *n* = 70) and recipients who received a transplant from a donor of a different race (DRT; *n* = 29) (Table 4). There were more Caucasian recipients in the SRT group compared with the DRT group (67% versus 14%; *p* < 0.001). There were more transplants with deceased donors in the DRT group compared with the SRT group (65% versus 30%) and fewer transplants with a living-related donor in the DRT group (21% versus 56%; *p* = 0.002). Consequently, the DRT group had higher eplet mismatched loads compared with the SRT group for class I (includes HLA-A,-B-C; mean difference in eplet load = 9; 95% CI 2–15; *p* = 0.007) and class II (includes HLA-DRβ1/DRβ345/DQβ1/DPβ; mean difference in eplet load = 8; 95% CI 1–16; *p* = 0.029) (Fig. 2). Interestingly, there was no difference in the mean eplet load when considering only mismatches for HLA-DRβ1 (*p* = − .573) (Fig. 2).

**Table 4:** Outcome based on pairing of donor and recipient race

Recipient race, *n* (%)	SRT^1^ (*n* = 70)	DRT^2^(*n* = 29)	*p* value
Caucasian	47 (67)	4 (14)	<0.001
African American	19 (27)	15 (52)	
Other	4 (6)	10 (34)	
Donor source, *n* (%)			
Deceased donors	21 (30)	19 (65)	0.002
Living-unrelated donors	10 (14)	4 (14)	
Living-related donors	39 (56)	6 (21)	
Induction treatment, *n* (%)			
Thymoglobulin	49 (70)	20 (69)	0.999
Daclizumab	4 (6)	3 (10)	0.413
Basiliximab	4 (6)	2 (7)	0.999
Alemtuzumab	1 (1)	1 (3)	0.502
Unknown^3^	12 (17)	3 (11)	0.542
HLA antigen mismatch, mean (SD)			
HLA class I (A,B,C) mismatch	3.2 (0.1)	4.3 (0.2)	<0.001
HLA class II (DR,DQ,DP) mismatch	2.9 (0.1)	3.9 (0.2)	0.002
Transplant outcome, *n* (%)			
de novo DSA	28 (40)	12 (41)	0.999
Rejection	25 (36)	11 (38)	0.823
Graft loss	15 (21)	4 (14)	0.575
Disease recurrence	9 (13)	3 (10)	0.999
Follow-up time (years)	5.9 (0,38)	6.3 (0.57)	0.557

^1^SRT: same race transplant

^2^DRT: different race transplant

^3^Unknown: no information on induction

### Incidence of de novo HLA-DSA

Post-transplant HLA antibody assessments were performed for 86 of the 110 (78%) transplanted patients. By end of follow-up, 59 of 86 patients (68%) were sensitized against HLA antibody. Moreover, 48 of the 86 patients (56%) had detectable antibodies directed against one or more donor antigens (de novo HLA-DSA). Of those who developed de novo HLA-DSA, 14 patients (27%) had infections and immunosuppression was reduced for 4 patients (8%) due to infection; medication non-adherence was reported for 10 patients (19%), and 2 (4%) had disease recurrences. Since sera were tested only at time of dysfunction, it was not possible to accurately determine when HLA-DSA developed; however, the time from transplantation to first detection of HLA-DSA ranged between 5 and 135 months (median time 50 months). The majority of de novo HLA-DSA was against HLA class II (47 were positive for HLA class II and 20 were positive for both HLA class I and class II antibody). There were more antibodies against HLA-DR and HLA-DQ antigens compared with all other antigens (*p* < 0.001). In an unadjusted model, the risk for development of de novo DSA was statistically significantly higher with greater total eplet loads for DR and DQ but not HLA-A, HLA-B, and HLA-C (Table 5). When only considering antibody verified eplets, the risk of developing de novo DSA was increased for all loci (supplemental Table 6A). After adjusting for all confounders, the risk of de novo HLA-DSA with increased eplet loads for class II remained statistically significant (RR = 1.02; 95% CI 1.00–1.03; *p* = 0.01). Similarly, the relative risk of developing de novo DSA was higher with increased HLA antigen mismatches (supplemental Table 6B).

**Table 5:** Crude^1^ association between total eplet mismatch and outcomes

Outcome	Total eplet MM^2^	N^3^	RR	95% CI	*p* value
de novo DSA	ABC	92	1.01	1–1.03	0.089
	DRβ1/3/4/5,DQβ1, DPβ1	82	1.02	1.01–1.03	< 0.001
	DRβ1/3/4/5	92	1.02	1–1.05	0.039
Rejection	ABC	102	1.01	1–1.03	0.111
	DRβ1/3/4/5,DQβ1, DPβ1	82	1.00	0.99–1.02	0.611
	DRβ1/3/4/5	110	1.01	0.98–1.03	0.604
Graft Loss	ABC	105	1.00	0.97–1.02	0.674
	DRβ1/3/4/5,DQβ1, DPβ1	82	1.01	0.99–1.03	0.568
	DRβ1/3/4/5	105	1.00	0.97–1.04	0.782

^1^Calculations from unadjusted models

^2^Total eplet MM: antibody verified and non-verified eplets

^3^N: Number of patients with available data

The incidence of de novo DSA development was not significantly different between SRT and DRT groups (44% versus 48%; *p* = 0.7; Table 4). There was no significant difference in class I and class II eplet mismatch load between DRT with de novo DSA (*n* = 12) compared with SRT with de novo DSA (*n* = 35) (supplemental Table 7). After adjusting for all confounders, the association between eplet mismatch for class I and class II molecules and de novo DSA development was also not significantly different between DRT and SRT group for class I (RR = 0.99; 95% CI 0.95–1.03; *p* = 0.7) and for class II (RR = 0.98; 95% CI 0.96–1.01; *p* = 0.4).

### Incidence of rejection based on eplet load

**Fig. 3:**
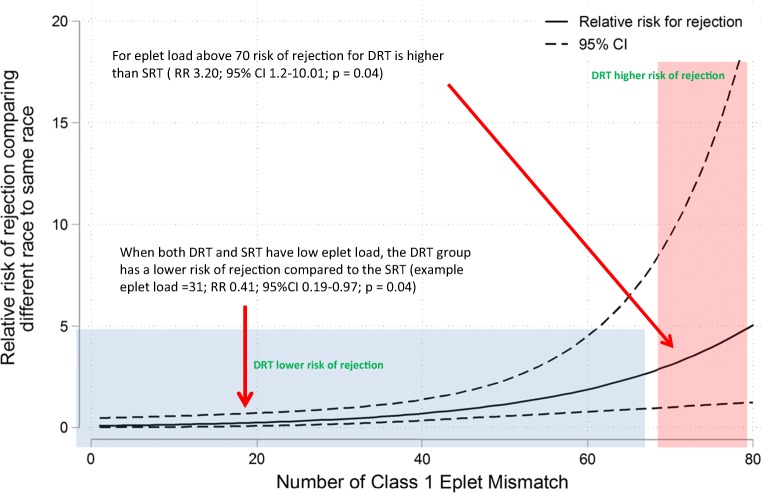
Relative risk of rejection with increasing class I eplet mismatch. After adjusting for donor and recipient age, gender, donor source, kidney allocation period, recipient diagnosis, and CPRA, the solid curve illustrates the increased association between eplet MM and race mismatch. Each point on the solid curve denotes the relative risk for rejection when comparing DRT and SRT at a certain class I eplet mismatch level. The dash lines denote the 95% confidence intervals. As the number of class I eplet mismatch increases, the association between the number of eplet mismatches and incidence of rejection increases. Number of patients with eplet load < 20, *n* = 21; 20–50 eplet load *n* = 70; eplet load > 50 *n* = 19

The incidence of rejection in the entire cohort was 38% (*n* = 42) and included CMR and AMR. The relative risk of rejection was not significantly higher as the number of total, antibody verified eplet loads and HLA-mismatched antigens increased (Table 5 and supplemental Table 6). The risk of rejection for the DRT group became significantly higher compared with the SRT group when the class I mismatched eplet load rose to ≥ 70 (RR = 1.05; 95% CI 1.01–1.08_;_*p* = 0.004; Fig. 3). The risk of rejection was not significantly increased for the DRT group compared with the SRT group with greater total eplet load for class II (RR = 0.94; 95% 0.88–1.01; *p* = 0.1).

### Graft loss based on eplet load

Graft loss was reported for 25 patients (23%) in this cohort; 22 of the 25 patients had de novo DSA, 14 (56%) had rejection prior to graft loss, and 5 patients (20%) had reports of medication non-adherence. As shown in Tables 4 and 5, we found no increased risk of graft loss based on total or antibody verified eplet loads at all loci (RR = 1; 95% CI 0.99–1.01; *p* = 0.7). Similarly, we found no association between graft loss and total or antibody verified eplet load differences in SRT versus DRT groups for all loci. There was also no correlation between antigen mismatch and graft loss.

## Discussion

The objective of this study was to investigate whether higher eplet mismatch loads between kidney transplant recipients and donors of a different race (DRT) negatively impacted transplantation outcome when compared with recipients who received a kidney from a donor of the same race (SRT). We performed a single center, retrospective analysis of 110 pediatric kidney transplant recipients of a first transplant, with a mean follow-up time of 5.8 years. We confirmed that higher eplet loads correlate with increased incidence of de novo HLA-DSA (Table 5 and supplemental Table 6). However, the risk of de novo HLA-DSA development, rejection, or graft loss did not differ significantly between DRT and SRT groups (Table 4). Additionally, only HLA class I eplet load greater than 70 resulted in a greater risk of rejection in the DRT group compared with the SRT group (Fig. 3).

The study was initiated as a cautionary response to recent studies suggesting the use of eplet mismatch load “thresholds” to select donors for transplantation. In a study by an Australian group, thresholds of less than 10 eplets for HLA class I and less than 30 eplets for HLA class II were used to allocate deceased donors for pediatric transplant candidates [20]. The racial characteristics of donors and recipients were not provided in this study. The US population is more heterogeneous than that of Europe and Canada [33, 34] and this has implications for the incidence of HLA alleles within each of these populations [35]. Data from the scientific registry of transplant recipients (SRTR) show that 65 to 70% of deceased and living donors from 2006 until 2017 were Caucasian compared with less than 20% of donors listed as African American, Hispanic, or other unspecified races [36]. Conversely, the racial makeup of pediatric transplant candidates on the waitlist is almost equal between the racial groups [36]. Our study shows that patients who were matched with a donor of a different race had higher eplet mismatch loads (Fig. 2), although not worse outcomes, compared with patients transplanted with a donor of the same race (Table 4). While the concept of eplet mismatch deserves serious consideration as a mechanism for improving transplantation outcome for pediatric transplant recipients, there is a need for larger studies, involving heterogeneous populations to determine optimal eplet thresholds if it is to be used for donor selection.

Since several studies have documented that a higher eplet mismatch load between donor and recipient is associated with poor transplant outcomes (RW.ERROR - Unable to find reference: 2591), it is important to understand the reason for the observed outcomes in the DRT group compared with the SRT group. An HLA molecule contains several eplets. As an example, in the HLA epitope registry (www.epregistry.com), 36 eplets are identified for HLA-A*01:01 allele (Fig. 1). Only 12 of the 36 eplets have reported HLA antibodies identified in patient sera (Fig. 1; antibody verified = yes). The remaining 24 eplets are called “non-antibody verified” eplets. Studies have shown that the immunogenicity of an eplet, defined as the ability of this antigenic configuration to elicit an immune response, is dependent on important physiochemical characteristics of the amino acids that make up the eplet [37]. These physiochemical properties include the electrostatic potential (polar and charged residues, and bonding interactions) and hydrophobicity of the amino acids [38]. This suggest that further stratification of the DRT cohort based on the properties of the mismatched eplets could identify those at greater risk for poor outcome.

We observed no significant difference in the eplet mismatch load between Caucasian and non-Caucasian patients. In their most recent publication, Wiebe and colleagues classified the eplet load threshold for HLA-DR and HLA-DQ molecules, into 3 risk categories (low = DR < 7 and DQ < 9, intermediate = DR ≥ 7 and DQ < 14 and high = DR 7–22 and DQ 15–31). In our study, the eplet load difference between the SRT and the DRT groups for HLA class II ranged between 1 and 16 with a mean eplet load difference of 8 (Fig. 2). These mismatched loads were within the intermediate range as determined in Wiebe et al. (HLA-DR 0–6 and HLA DQ 9–14). Therefore, the DRT cohort is not at a significant disadvantage compared with the SRT group when considering HLA class II mismatched antigens. The use of HLA class II eplet mismatch load rather than HLA class I eplet load, across racially diverse transplant populations, may allow more equitable distribution of donor organ.

The incidence of de novo HLA-DSA was 56% in this cohort, which is higher than the rates of 17 to 34% that have been reported in previous pediatric studies [39–42]. While these studies evaluated the incidence of de novo DSA in the entire study cohort [15], we determined our rate based only on patients who presented with dysfunction. Therefore, this increased incidence is from a biased cohort. Steggerda et al. report a similar incidence of 56% for de novo DSA post-transplantation in cohorts with suspected antibody-mediated rejection [14, 43, 44]. Development of de novo DSA was associated with infections which resulted in a reduction in immunosuppression. Furthermore, in line with most studies [45, 46], we identified a higher incidence of HLA-DQ antibody. Interestingly, Mallon et al. [38] found that electrostatic potential disparities are highest among HLA-DQ molecules. McCaughan et al. [45] further characterized a high-risk eplet mismatch in HLA-DQ7 associated with increased incidence of HLA antibody development. This data further supports eplet mismatch load analysis for HLA class II molecules.

In our study, we did not compare transplant outcomes based on donor type, but observed that the DRT group included more deceased donor transplant recipients, yet suffered no increased incidence adverse post-transplant outcome (Table 4). However, the choice between a less well-matched living donor and a well-matched deceased donor is controversial. Marlais et al. [47] reported that living donor transplants, regardless of degree of HLA matching, had better outcomes than a well-matched deceased donor organ. A follow-up study by Opelz et al. showed better outcome with well HLA-matched deceased donors compared with living donor transplants who had more mismatches at HLA-A-B and DR loci [9]. The use of eplets rather than antigen does provide additional granularity for matching HLA antigens between donor and recipient. Many eplets are found on more than one HLA molecule. Using the example of HLA-A*01:01 allele, 32 of the 36 eplets of HLA-A*01:01 are also present on HLA-A*03:01 (Fig. 1). The shared eplets between different HLA antigens increases the pool of potential matched antigens and may reduce antibody development against antigens that share common eplets.

We did not find an association between eplet load and graft loss in this pediatric cohort (Tables 2 and 3). Wiebe et al. reported a greater incidence of graft loss in non-adherent transplant recipients with graft loss and DR eplet load > 10 [48]. In our cohort, only 5 patients had report of medication non-adherence associated with allograft loss, a significantly lower number compared with Wiebe et al., which may contribute to the difference in finding between our study and this group [48]. Furthermore, tacrolimus was used as standard treatment in this cohort, while a significant number of patients were treated with cyclosporine in the Canadian study [49]. This group later showed better outcome in patients with higher eplet load treated with an appropriate dose of tacrolimus compared with cyclosporine [49]. In a retrospective analysis of pediatric heart transplant recipients, Sullivan and co-authors also reported an association between HLA eplet load and graft loss [50]. Importantly, in this study, the median graft survival time was 13.5 years; with a graft survival time for patients with highest eplet mismatch loads greater than10 years. Therefore, it is not surprising that we found no correlation between increased eplet mismatch load and graft loss in our cohort with a much shorter median follow-up time of 5 years.

The limitations of this study are characteristics of retrospective studies, and include a small sample size and missing data. We obtained eplet mismatched loads using intermediate resolution typing since only antigen level typing is reported for solid organ transplant recipients. Similar to several other studies [27, 45, 51], we have used the most common allele to obtain eplet load in HLA Matchmaker. Additionally, the study did not include analysis of HLA-DQA and DPA eplets as more than 50% of the patients and donors were missing DQA typing results. Although DQA could be inferred using HLA-DRB and HLA-DQB associations and patient race, this could introduce addition error in the analysis. Furthermore, of 286 HLA class II eplets identified, the registry lists 25 DQA (9% of total HLA class II eplets; only 3 verified with specific antibodies) and 15 DPA eplets (5% of total HLA class II eplet; none of which are antibody confirmed) [52]. This does not represent a significant increase in the total eplet count. A third limitation is the missing data for donor race. Nevertheless, our results support previous observations that show a correlation between incidence of de novo DSA and rejection with increased eplet loads.

Optimizing outcomes for pediatric transplant recipients includes optimal immunological matching in order to reduce de novo DSA formation, rejection episodes, and need for increased immunosuppression. This study demonstrates that eplet mismatch analysis is a more sensitive tool to predict some but not all outcomes in this pediatric transplant cohort, but one must use caution in including this analysis for donor selection [53]. Eplet mismatch load analysis could assist in selection of the best living donor when several potential donors are available. This tool could also be used in a complementary fashion, to determine better post-transplant monitoring strategies as well as immunosuppression regulation. For example, careful reduction in immunosuppressive therapy or use of less aggressive treatment may be considered for donor/recipient pairs with lower eplet loads. Alternatively, we found no significant difference between eplet mismatch load for HLA class II based on racial distribution. Therefore, using the eplet load mismatch for HLA class II could be a better approach to select a donor that may be immunologically suitable, while not eliminating options for patients who have different racial backgrounds compared with the donor pool. Importantly, we found that despite the higher eplet mismatch load in the DRT group, the outcomes in the two groups were comparable notwithstanding the limitations of the study. Larger, multicenter, prospective studies are needed to carefully assess the effect of epitope-based allocation.

## Electronic supplementary material

ESM 1 (DOCX 14 kb)

ESM 2 (DOCX 17 kb)

## Abbreviations

AMR: Antibody-mediated rejection
CMR: Cell-mediated rejection
cPRA: Calculated percent reactive antibody
HLA-DSA: Donor specific HLA antibody
FCXM: Flow cytometric crossmatch
KAS: Kidney allocation system
MFI: Mean fluorescent intensity
DRT: Different race transplants
SRT: Same race transplants
SRTR: Scientific registry for transplant recipients
