# Correction to: Eplet mismatch analysis and allograft outcome across racially diverse groups in a pediatric transplant cohort: a single-center analysis

**DOI:** 10.1007/s00467-019-04444-y

**Published:** 2019-12-11

**Authors:** Mary Carmelle Philogene, Anita Amin, Sheng Zhou, Olga Charnaya, Renato Vega, Niraj Desai, Alicia M. Neu, Cozumel S. Pruette

**Affiliations:** 1 Department of Medicine, Johns Hopkins School of Medicine, 2041 E. Monument Street, Baltimore, MD 21205 USA; 2 Immunogenetics Laboratory, 2041 E. Monument Street, Baltimore, MD 21205 USA; 3 Department of Neurology, University of North Carolina School of Medicine, 115 Mason Farm Road, Chapel Hill, NC 27599 USA; 4 Department of Surgery, Johns Hopkins University School of Medicine, 720 Rutland Ave Turner 34, Baltimore, MD 21205 USA; 5 Department of Epidemiology, Johns Hopkins School of Public Health, Baltimore, MD USA; 6 Department of Pediatric Nephrology, Rubenstein Child Health Building, Johns Hopkins University School of Medicine, 200 N. Wolfe Street, Baltimore, MD 21287 USA

**Correction to: Pediatric Nephrology**

10.1007/s00467-019-04344-1

The original version of this article unfortunately contained a mistake. In the third paragraph of “Discussion,” two references were missing. The correct wording is “Since several studies have documented that a higher eplet mismatch load between donor and recipient is associated with poor transplant outcomes [50, 54], it is important to understand the reason for the observed outcomes in the DRT group compared with the SRT group.”

In addition, reference [54] is missing in the original. Please find the two cited references below.
