# Supplementary material for: Eplet mismatch analysis and allograft outcome across racially diverse groups in a pediatric transplant cohort: a single-center analysis
Source: Pediatr Nephrol. 2019 Oct 10;35(1):83–94. doi: 10.1007/s00467-019-04344-1 (PMC6901410; doi:10.1007/s00467-019-04344-1)
Supplement: Supplementary file 1 — (DOCX 14 kb) [file 467_2019_4344_MOESM1_ESM.docx]

| **Supplemental Table 6A. Crude Association between Antibody Verified Eplet mismatch and Outcomes^1^** | | | | | |
| --- | --- | --- | --- | --- | --- |
| **Outcome** | **Antibody Verified Eplet MM** | **N** | **RR** | **95% CI** | **p-value** |
| ***de novo* DSA** | ABC | 90 | 1.06 | 1.02-1.1 | 0.008 |
|  | DRβ1/3/4/5,DQβ1 | 89 | 1.08 | 1.03-1.13 | 0.001 |
|  | DRβ1/3/4/5 | 89 | 1.11 | 1.02-1.2 | 0.012 |
|  |  |  |  |  |  |
| **Rejection** | ABC | 92 | 1.04 | 0.99-1.09 | 0.155 |
|  | DRβ1/3/4/5,DQβ1 | 89 | 1.04 | 0.98-1.11 | 0.180 |
|  | DRβ1/3/4/5 | 89 | 1.05 | 0.98-1.11 | 0.197 |
|  |  |  |  |  |  |
| **Graft Loss** | ABC | 94 | 0.97 | 0.89-1.04 | 0.391 |
|  | DRβ1/3/4/5,DQβ1 | 91 | 1.03 | 0.93-1.13 | 0.512 |
|  | DRβ1/3/4/5 | 91 | 1 | 0.88-1.16 | 0.936 |

| **Supplemental Table 6B. Crude Association between Antigen Mismatch and Outcomes^1^** | | | | | |
| --- | --- | --- | --- | --- | --- |
| **Outcome** | **Antigen MM** | **N** | **RR** | **95% CI** | **p-value** |
| ***de novo* DSA** | ABC | 92 | 1.2 | 1.00-1.43 | 0.048 |
|  | DRβ1/3/4/5,DQβ1 | 82 | 1.26 | 1.06-1.49 | 0.008 |
|  |  |  |  |  |  |
| **Rejection** | ABC | 102 | 1.19 | 0.99-1.44 | 0.064 |
|  | DRβ1/3/4/5,DQβ1 | 81 | 1.19 | 0.97-1.47 | 0.098 |
|  |  |  |  |  |  |
| **Graft Loss** | ABC | 105 | 1.07 | 0.84-1.36 | 0.577 |
|  | DRβ1/3/4/5,DQβ1 | 82 | 1.26 | 0.92-1.31 | 0.158 |
|  |  |  |  |  |  |

^1^Calculations results from unadjusted models
