# Supplementary material for: Eplet mismatch analysis and allograft outcome across racially diverse groups in a pediatric transplant cohort: a single-center analysis
Source: Pediatr Nephrol. 2019 Oct 10;35(1):83–94. doi: 10.1007/s00467-019-04344-1 (PMC6901410; doi:10.1007/s00467-019-04344-1)
Supplement: Supplementary file 2 — (DOCX 17 kb) [file 467_2019_4344_MOESM2_ESM.docx]

| **Supplemental Table 7A: Eplet load in DRT versus SRT associated with Outcomes** | | | | | |  |
| --- | --- | --- | --- | --- | --- | --- |
|  | **Class I Eplet MM** | | | **Class II Eplet MM** | | |
|  | **SRT** | **DRT** | ***p*** | **SRT** | **DRT** | ***p*** |
| ***de novo* DSA** | 30 | 38 | 0.861 | 33 | 36 | 0.360 |
| **No *de novo* DSA** | 27 | 37 |  | 20 | 31 |  |
| **Rejection** | 29 | 44 | 0.600 | 28 | 29 | 0.350 |
| **No rejection** | 28 | 34 |  | 23 | 35 |  |
| **Graft loss** | 24 | 37 | 0.592 | 27 | 38 | 0.999 |
| **No graft loss** | 30 | 37 |  | 24 | 32 |  |
| **Table 7B: Antigen Mismatch in DRT versus SRT associated with Outcomes** | | | | | |  |
|  | **Class I Eplet MM** | | | **Class II Eplet MM** | | |
|  | **SRT** | **DRT** | ***p*** | **SRT** | **DRT** | ***p*** |
| ***de novo* DSA** | 3 | 4 | 1 | 3 | 4 | 1 |
| **No *de novo* DSA** | 4 | 4 |  | 4 | 4 |  |
| **Rejection** | 4 | 5 | 1 | 3 | 4 | 1 |
| **No rejection** | 3 | 4 |  | 3 | 4 |  |
| ^1^SRT: same race transplant |  |  |  |  |  |  |
| ^2^DRT: different race transplant |  |  |  |  |  |  |
| ^3^Unknonwn: no information on induction | |  |  |  |  |  |
